# Limitations of the Human Reference Genome for Personalized Genomics

**DOI:** 10.1371/journal.pone.0040294

**Published:** 2012-07-11

**Authors:** Jeffrey A. Rosenfeld, Christopher E. Mason, Todd M. Smith

**Affiliations:** 1 Division of High Performance and Research Computing, University of Medicine & Dentistry of New Jersey, Newark, New Jersey, United States of America; 2 American Museum of Natural History, Sackler Institute for Comparative Genomics, New York, New York, United States of America; 3 Department of Physiology and Biophysics, Weill Cornell Medical College, New York, New York, United States of America; 4 The HRH Prince Alwaleed Bin Talal Bin Abdulaziz Alsaud Institute for Computational Biomedicine, Weill Cornell Medical College, New York, New York, United States of America; 5 PerkinElmer, Seattle, Washington, United States of America; Seoul National University College of Medicine, Republic of Korea

## Abstract

Data from the 1000 genomes project (1KGP) and Complete Genomics (CG) have dramatically increased the numbers of known genetic variants and challenge several assumptions about the reference genome and its uses in both clinical and research settings. Specifically, 34% of published array-based GWAS studies for a variety of diseases utilize probes that overlap unanticipated single nucleotide polymorphisms (SNPs), indels, or structural variants. Linkage disequilibrium (LD) block length depends on the numbers of markers used, and the mean LD block size decreases from 16 kb to 7 kb,when HapMap-based calculations are compared to blocks computed from1KGP data. Additionally, when 1KGP and CG variants are compared, 19% of the single nucleotide variants (SNVs) reported from common genomes are unique to one dataset; likely a result of differences in data collection methodology, alignment of reads to the reference genome, and variant-calling algorithms. Together these observations indicate that current research resources and informatics methods do not adequately account for the high level of variation that already exists in the human population and significant efforts are needed to create resources that can accurately assess personal genomics for health, disease, and predict treatment outcomes.

## Introduction

A primary goal of the human genome project was to produce a high quality DNA sequence that could serve as a common reference for understanding the genetic basis of health and disease. The reference sequence has been a guiding principle for the development of a vast array of reagents, arrays, genotyping assays, computational tools, and clinical resources. Moreover, the reference sequence is the foundation for databases and bioinformatics algorithms that are used to define target regions for resequencing, perform genome wide association studies, or measure inter-species conservation. Thus, the reference sequence has become essential for clinical applications, and is used to determine alleles for risk, protection, or treatment-specific response in human disease [1]. Yet, the current reference sequence, being based on a limited number of samples, neither adequately represents the full range of human diversity, nor is complete [2], [3].

Because so much work is currently based on the concept of a standardized reference sequence, we have evaluated the extent to which our growing knowledge of human genome variation should alter this paradigm. New data emerging from the 1000 Genomes Project (1KGP) [4] and public release of genomes from Complete Genomics (CG) [5] have dramatically increased the numbers of known genetic variants by tens of millions [6]. Using the 1KGP and CG genome datasets, we have evaluated several genomics tools and assays that have been developed with the reference sequence. In addition to identifying a high frequency of confounding issues with microarrays, multiple instances where bioinformatics programs rely on invalid assumptions to underestimate variability or possibly misidentify the functional effects of mutations were found. Additional comparisons of the inter- and intra-population variance within the CG and the lower coverage 1KGP experiments identified a striking degree of difference (>19%) between the variants called on the same genomes. These findings have implications for the study of human variation and medical genetics, and resolving and correcting these discrepancies will be essential for creating the era of personalized medicine. Importantly, the results of these studies are stored in dbSNP and are regarded as the true lists of variants for the genotyped individuals. This is extremely problematic due to the high false-positive rates of the technologies and the diversity of results [4]. Further development of genotype calling techniques and methods for validation are required in order to make these data usable and reliable.

## Results

### Most Microarray Probes are Confounded by Genetic Variation

Microarrays have been one of the most utilized tools in genetic research and are the basic platform for Genome-Wide Association Studies (GWAS). These arrays contain millions of DNA probes that are used to determine the genotypes of polymorphic loci. The optimal genotyping capacity is predicated on two basic assumptions concerning genomic sequences. First, the genotyped samples are completely identical at all locations within the probe, except for the targeted SNP. Second, the SNP must be one of the two variants for which the array was designed as variants are assumed to be biallelic. If either of these two conditions are violated, then the microarray probe will not function as well, or at all, and lead to false negative or false positive results for some individuals [7].

**Figure 1:**
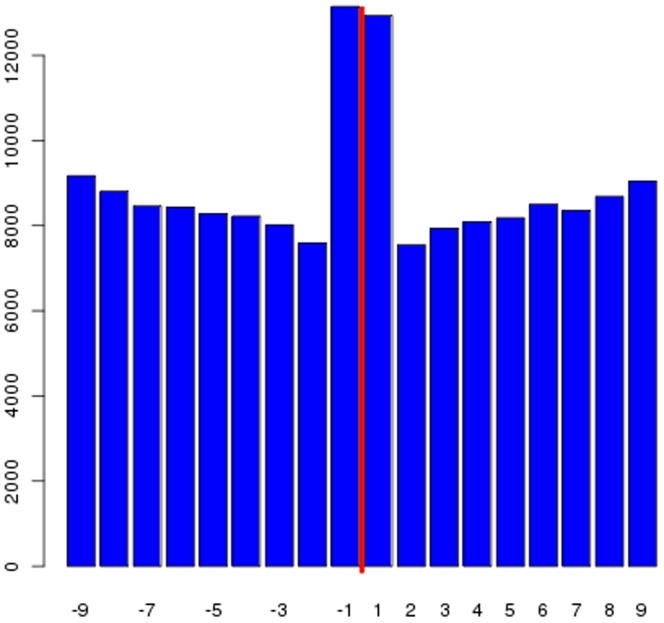
Location of SNPs relative to the probed base in microarrays. Histogram showing the number of SNPs in upstream and downstream positions relative to the probed SNP on the Illumina1 M array. The red line indicates the location of the probed SNP.

**Figure 2:**
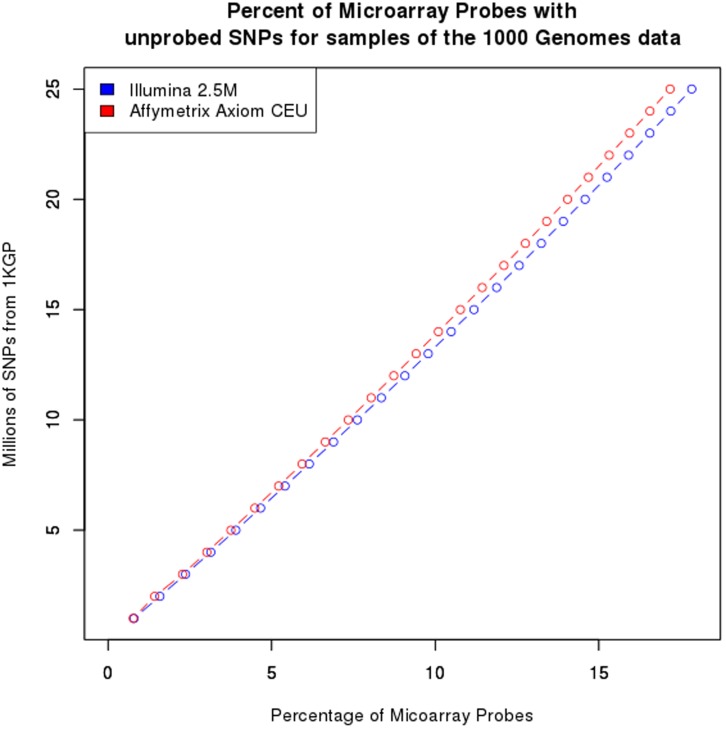
Problematic probes on microarrays. The number of probes on the Affymetrix Axiom CEU (blue) and Illumina 2.5 M(red) arrays that are found to contain an un-probed SNP for sub-samples of the 1KGP SNPs.

Using the 1KGP dataset (25 million single nucleotide polymorphisms (SNPs) from 629 individuals), we evaluated the extent to which these assumptions are violated for each of four commonly used microarray platforms, the Illumina Human Omni1M-quad, Illumina Omni 2.5 M, Affymetrix 6.0, and the Affymetrix Axiom CEU array, which takes into account new knowledge concerning variation within the genome [8]. For each probe, we tested whether there were any SNPs or indels detected in the 1KGP data within 10bp of the targeted SNP on either the 5′ or 3′ side of the probe. We also tested whether the probe was contained within an annotated structural variant (SV). Overall, a substantial percentage of probes (51%) are affected by one or more of these problems (Table 1). The summary columns indicate the number of probes that have any of the listed deficiencies and certain probes are problematic for more than one reason. It is also worth noting that the distribution of untyped SNPs surrounding the probed SNP is reasonably constant with the exception of a higher number of SNPs the +1 and -1 locations (Figure 1B), because base differences closer to the probe base are more likely to affect hybridization than those further away. The higher level of variants at the +1 and -1 locations is likely due to the potential mis-mapping of reads and probes to the genome [9], [10]. In order to determine whether we have found the majority of problematic probes, we took subsamples of the 1KGP SNPs and counted how many probes on arrays were affected (Figure 2). We found that as the number of SNPs sampled increases, the number of probes with un-probed SNPs increases and the plot shows no sign of flattening. If the plot was reaching an asymptote, then we would have found most of the problematic probes and the rest of the probes could be confidently utilized. Thus, when all of the SNPs have been found in the human genome, there will be few if any locations where a microarray probe could be confidently utilized across all populations.

**Table 1:** List of Probes on Common array platforms adversely affected by variants detected through sequencing and not probed on the array.

		Number of Microarray probes overlapping				
Array	Number ofautosomalSNP probeson the array	UnprobedSNPs	Indels	StructuralVariants	Total number ofprobes affected byeither un-probedSNPs or Indels	Total number of probesaffected by either un-probedSNPs, Indels or StructuralVariants
Affymetrix 6.0	894,240	119,341 (13%)	7,720 (1%)	359,592 (40%)	125,785 (14%)	434,702 (49%)
Affymetrix Axiom CEU	607,555	100,339 (17%)	11,983 (2%)	247,944 (41%)	111,150 (18%)	312,736 (51%)
Illumina 1 M	940,876	144,431 (15%)	9,996 (1%)	378,809 (40%)	153,192 (16%)	469,417 (50%)
Illumina 2.5 M	2,390,395	379,271 (16%)	35,494 (1%)	944,510 (40%)	410,355 (17%)	1,191,584 (50%)

Next, we examined polyallelic SNPs, where there are more than 2 possible alleles at a locus. The 1KGP data contained 496 loci over 629 genomes, whereas the Complete Genomics (CG) data contained 61,153 loci in just 69 genomes, representing a 123-fold increase in detection. Polyallelic SNPs are more difficult to investigate because many variant calling pipelines are biased toward biallelic SNPs [11], [12], which in part accounts for the large observed discrepancy. There were also a substantial number of positions where an individual is heterozygous, and yet neither base matched the reference. Such “heterozygous, dual non-reference” calls occur an average of 1,189 times per genome (sd = 288).

We then compared the “heterozygous, dual non-reference” SNP locations to lists of the probe bases on the standard microarrays and found thousands of instances where the microarray probe assumed a biallelic context but is instead polyallelic (Table S1). This presence of more than 2 alleles at a site has been found to be problematic for many different genotyping platforms [13],[14], and these types of errors have also been shown to affect variant calling accuracy in exome sequencing [15]. We observed 2,928, 1,993, 2,918, and 2,118 probes were affected on the Affymetrix 6.0, Axiom CEU, Illumina 1 M, and Illumina 2.5 M arrays, respectively.

The impact of the above issues is that they can confound GWAS studies due to false homozygous or negative calls. Hence, we next sought to examine which of these problematic probes appear in published GWAS studies. 34% of the studies (1,708/4,972 from the UCSC genome browser) have significant hits with a microarray probe affected by neighboring un-probed SNPs, SVs, indels or polyallelic SNPs (Table S1). Though many of these studies take great care to avoid non-random genotyping errors, current methods to detect such errors would not incorporate these types of changes. Thus, we caution that replication of future GWAS studies should attempt to be robust to both population stratification as well as genomic variation, by using additional probes and/or orthogonal technologies for genotyping top candidates. A list of all microarray probes that were found to be problematic along with the reasons for their deficiency are listed in Table S2.

### Genes and Exons with Multiple Variants

Widely used bioinformatics tools for examining the effect of a specific mutation on a protein’s structure, such as PolyPhen [16] or SIFT [17], also depend on the reference sequence. Such programs take the reference sequence and a polymorphism and predict the likely effects of the variant on the modified protein’s structure and functional capability. While the ability of these programs to fully predict the functional consequence of a mutation is not perfect [18], [19], they are still widely utilized for variant stratification and prioritization. Yet, these programs were designed to examine only one deleterious variant at a time, and thus, complex intra-protein interactions between multiple variants are lost, such as compensatory, additive, or exacerbating effects [20]–[22].

We utilized the high-depth CG genomes to evaluate the extent to which there are multiple SNPs within an exon or the full coding regions of a gene. For each individual, there is an average of 6,077 genes (sd = 570) having multiple SNPs and an average of 3,320 (sd = 341) individual exons having multiple SNPs. The vast majority of exons with multiple SNPs only have two SNPs, but there are some exons with a larger number of SNPs. BRCA1, one of the most well studied breast cancer genes, contains numerous genetic variants. In the CG data 36 of 69 individuals have multiple (2–5) non-synonymous variants within their BRCA1 gene.

### Intra-Population Variation and Linkage Disequilibrium

Haplotype blocks can, in theory, reduce genotyping measurements by using one SNP to “tag” other SNPs [23], [24]. Using HapMap [25] data, large regions of linkage disequilibrium (LD) have been determined for different human populations to create haplotype blocks that organized SNPs into co-segregating groups. The larger number of SNP loci provided by the 1KGP allowed us to investigate how the number of loci affects LD block size. The CEU (Caucasian American) datasets from HapMap and 1KGP were used to compare the respective LD and homozygosity of genomes. Both datasets contain 90 individuals and many of the samples are shared between the two studies. Because LD calculations are sensitive to missing data, the two datasets were trimmed to only include SNP loci that had genotype calls of at least 99%. This resulted in the HapMap set having 2,297,650 SNPs and the 1KGP set having 13,289,610 SNPs. For the HapMap set, the mean LD block was 16.4 kb in size (sd = 27.4) whereas the 1KGP dataset had a mean LD block size of 7.0 kb (sd = 15.7 kb). Thus, by increasing the number of markers used, the mean LD block decreased in size by half.

**Figure 3:**
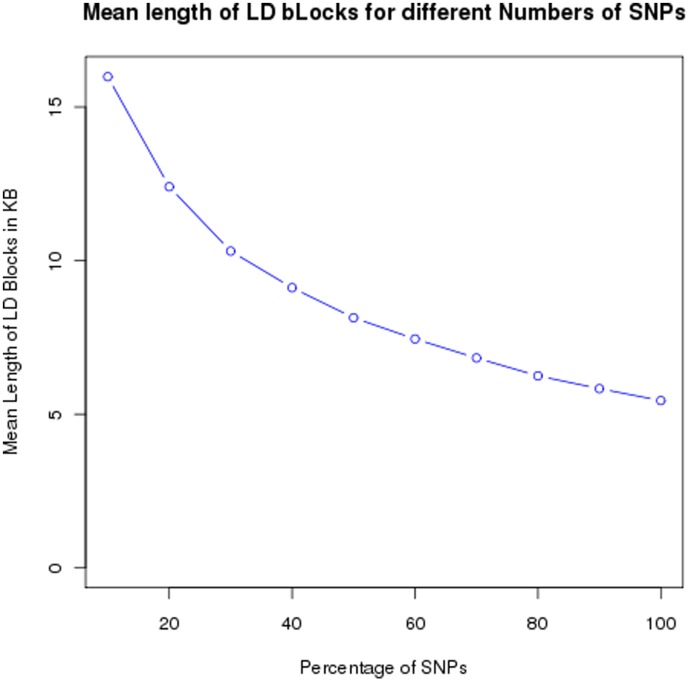
LD block lengths. The mean length of LD blocks as the number of genotyped markers increases.

**Figure 4:**
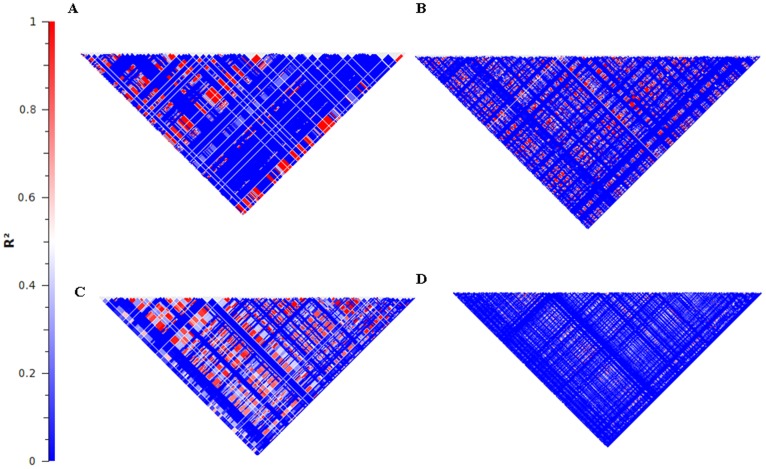
The LD block structure of two genes for the HapMap data and the 1KGP data. **A.** The BRCA1 gene using the HapMap data. **B.** The BRCA1 gene using the 1KGP data. **C.** The JAK2 gene using the HapMap data. **D.** The Jak2 gene using the 1KGP data.

To further determine the extent to which both the number of SNPs and the number of individuals analyzed affected LD calculations, we separately selected random samples of different percentages of individuals and SNPs from the 1KGP data for chromosome 20. As the number of SNPs increased (with a constant number of individuals), the average size of an LD block decreased from 16 kb (sd = 26 kb) to 5.4 kb (sd = 13 kb) (Figure 3). Based upon the figure, LD block size appears to be reaching an asymptote, but the size will continue to decrease as the number of identified SNPs increases. A clinically relevant example of how the decreased LD lengths can affect impact SNP-tagging is observed in the BRCA1 and JAK2 cancer genes (Figure 4 and Table 2). The HapMap data show extensive LD throughout the genes, whereas 1KGP data show many fewer strong blocks of LD. In particular, from the HapMap data, each gene, has four blocks, while from the 1KGP data, BRCA1 has seven haplotype blocks and JAK2 has 17 haplotype blocks.

**Table 2:** A comparison of the Linkage disequilibrium patterns of BRCA1 and JAK2 using HapMap and 1000 Genomes data.

Gene	Number of Blocks/total sizeof Blocks from HapMap	Mean size of Blocksfrom HapMap	Number of Blocks/total sizeof Blocks from 1KGP	Mean size of Blocksfrom 1KGP
BRCA1	4/73,185 bp	18.3 kb	7/75,015 bp	10.7 kb
JAK2	4/132,544 bp	33 kb	17/131,306 bp	7.7 kb

While it is understood that increasing variation will decrease LD block size, the impact of increased variation has not been documented. This decreased LD has strong implications for the diagnostic genotyping of these genes and association studies [26]. Regions previously presumed to be in LD [27], [28] can no longer be automatically considered strongly linked, and ideally the entirety of the gene should be considered for clinical work. For example, previous work on JAK2 was based on 92 haplotypes that were presumed tagged by individual SNPs [29], including one SNP that tagged the JAK2 46/1 haplotype and correlated with essential thrombocythemia [30]. Similarly, the finding that individual SNPs can tag BRCA1 haplotypes [31], [32] needs to be re-evaluated in light of the multiple haplotypes.

An additional measure used for studying population variation is the presence of runs of homozygosity (ROH). Individuals from a homogenous populations share similar chromosomal segments, and therefore, many contiguous loci across the individuals’ genomes would be homozygous. Conversely, individuals from admixed populations show less homozygosity in their genome. This homozygosity is investigated using SNP data and a run of homozygous (ROH) SNPs [33]. Such ROHs are only based upon the markers that are genotyped and the homozygosity of the intervening regions is assumed, but not truly known. We found that for the HapMap data, there is an average of 70.2 MB (sd = 16.7 MB) of homozygous sequence per genome, while in the 1KGP data, there was only an average of 13.2 MB (sd = 11 MB) of homozygous sequence per genome (see methods). This decrease in observed homozygosity reflects the increased resolution of the data and shows that our estimates of homozygosity have previously been far too high.

### Comparison of the 1000 Genomes and the Complete Genomics Datasets

**Figure 5:**
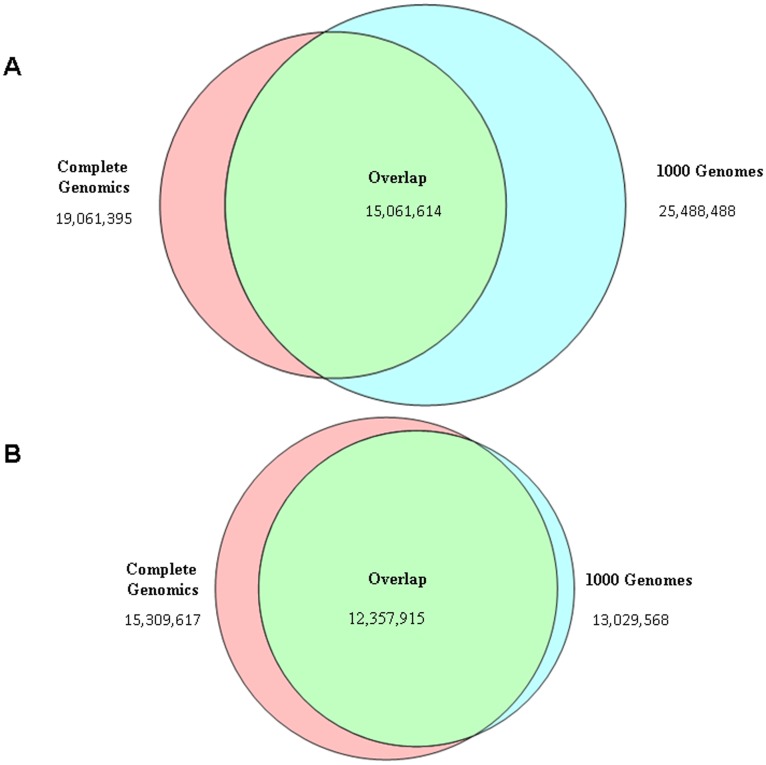
Venn diagrams illustrating the overlap in SNP calls between the 1KGP and CG. **A**. For the full call sets. **B**. For the matched set of 32 genomes.

The variant calls produced by 1KGP and CG represent two fundamentally different approaches to interrogating the variation across human genomes. The 1KGP project aims to sequence 2,500 low-coverage genomes, and the release we examined contained genotypes for 629 genomes. In contrast, the CG set contains 69 high-depth fully sequenced genomes. Since these approaches are so distinct, it is worth comparing the overlap of SNP calls between the two sets (Figure 5A). While a large number of SNPs (∼14 million) that are shared by both sets, 21% of the SNPs are unique to one of the sets. Part of the large difference could be due sampling differences; while both projects are sequencing HapMap individuals, there is not a complete overlap between the samples that were sequenced. Hence, the analysis was repeated with the 32 genomes that are shared by the two projects. Because the 1KGP sequencing is at a low depth, it will miss variants that should be detected in the higher coverage CG sequencing. The CG SNPs for these individuals should form a super set of those SNPs detected by 1KGP. Surprisingly, a substantial number (19%) of SNPs are still unique to one of the two platforms (Figure 5B) when comparing the same genomes. This difference is much greater than would be expected and is likely due to differences between data collection and analysis methods in the CG and 1KGP projects. Specifically, the CG genomes were genotyped individually without any reliance on other genomic sequences. Due to the low-coverage of the sequencing in the 1000 Genomes Data [4], multiple genomes were genotyped together and variation data was imputed between genomes. These two approaches are highly distinct from each other, and further validation using orthogonal methods will be required to evaluate which technique is more accurate.

## Discussion

Through efforts like the 1KGP and CG public data releases, we are getting a new view that human variation much more extensive than previously thought. These data also expose several shortcomings of current microarray tools and alter the view of some basic tenets of the allelic variance of the human genome.

While genotyping arrays have been a main tool in genetic studies for almost two decades, only now can we observe that current arrays will do an imperfect job measuring variation in randomized populations due to the greater than anticipated extent of probe-affecting variation. The regions near known SNPs have been assumed to be largely free of indels, SNPs, and SVs, and also biallelic, but all of these assumptions are incorrect for a large fraction of probes used in most genotyping platforms. The largest percentage of the probes is affected by structural variants, which cover a substantial percentage of the genome, ranging from insertions and deletions to large-scale tandem repeats and copy number variants. When a probe matches one of these regions, the actual location that is interrogated in the genome is ambiguous. In individuals lacking the SV, the reference location is interrogated, while in individuals with the SV, the variant location is interrogated. This inconsistency will produce variable results between individuals. We acknowledge that neither 1KGP data nor arrays are perfect and therefore the exact list of probes that are found to be potentially problematic will always be a moving target. Nevertheless, the overall counts and distribution of problematic probes would be highly similar if the 1KGP data were error-free.

As the number of questionable probes increases with the number of SNPs identified, it is doubtful that a “perfect” microarray or set of “population-specific” microarrays could be constructed based on the variation found in 1KGP, because only a small fraction of the variability of the human genome will be found. Even when the 1KGP is done, it will only have sampled 2,500 people, representing a very small percentage of people on the planet. Hence, sequencing will likely always be a more robust method to assess known and unknown variation in genomes.

Similar to shortcomings in large-scale microarray-based genotyping, functional predictions based on simple categorization of variants as synonymous or non-synonymous is limited. When multiple SNPs within a gene or an exon exist, the variants can act together [34], [35], and it is unknown whether these mutations are compensatory, additive, benign, or multiplicative. The only way to get a clear picture of a gene’s mutational burden is to examining the variants together [36]. Thus, we propose that it would be more useful to give a gene-wide call of synonymous or non-synonymous and that variant-calling algorithms should be modified or expanded to examine these interactions.

Other assumptions about human genetics, developed through previous scans of genomic variation, are also being re-evaluated as we collect more data. The average LD length, often used to tag SNPs and impute variation, decreases in size with increasing numbers of variants. As LD length distributions are Poisson, the number of blocks that are shorter than the average increases significantly with increasing numbers of variants. Similarly, bioinformatics programs and analyses that use assumptions based on HapMap [37], [38], or other early measurements, to filter data or report on functional consequences, will likely underreport observations and will need to be revised.

Clearly, data collection and analysis are integrally connected. This work demonstrates that the best approaches for assessing global variation in the human population at both the data collection and analysis phases are at an early stage. Methods are still being developed and the best way to make global measurements is under debate. As evidenced by the 1KGP and CG data sets, each set contains similar numbers of SNPs (1KGP = 25,488,488, CG = 19,154,014), yet the two sets are clearly different in the numbers of genomes represented and average base coverage per genome. Hence, it is worthwhile considering the economic costs and benefits of the deep individual or shallow population approaches. The 1KGP approach, using low coverage sequencing on many genomes, identified more SNPs and is on course to identify many more SNPs when the expected 2,500 genomes from a broad spectrum of populations are completed. The high coverage CG approach on a few genomes has produced fewer SNPs but more detailed information for each genome. For example, thousands of polyallelic SNPs were identified within the CG data set, but were almost completely missed by 1KGP. One reason for this is that the low coverage per-genome of the 1KGP dataset requires pooled genotyping, which tends to bias against rare or singleton variations. Additional factors for this disparity include annotation techniques. The 1KGP variant calling pipeline discards tri- and tetraallelic variants as errors, because alleles are either assumed to be biallelic or simply do not occur with high enough frequency in pooled data.

The current human population is over seven billion and growing. New mutations have been accumulating for over 5000 generations at the rate of between one and 100 mutations per generation [39] so the possibility exists that all SNPs compatible with life are represented in the entire population. When compensatory mutations are considered, genomic diversity can be even greater because a variant that would be fatal within one population could be entirely benign in another population. As such, the human genome reference sequence, while extraordinarily useful, has several shortcomings that need to be addressed to improve molecular diagnostic applications. Groups like the Genome Reference Consortium [40] and the 1KGP are developing a more complete and accurate reference. Other groups are proposing the use of specialized family references [41], or an ancestral allele reference [42]. However, new challenges are created with updating research tools and results to reflect the changes. Until such challenges are resolved, clinical utility of WGS will be limited to pre-ordained regions of the genome, with the caveat that tests will have low-level, uncertain false negative rates.

Moreover, in applications like tumor profiling, gene expression, or other functional genomics assays, a single reference sequence can be problematic. For a cancer genome, the best reference genome to which tumor data should be aligned is a matched normal genome of patient. This is the only way to be confident that driver mutations or rearrangements are novel in the tumor and not present in normal cells. In the case of RNA assays, cDNA reads should be mapped to the samples’ genomes from which the RNA was isolated. Some regions with known high variability, like the MHC, already have alternative assemblies because a single reference sequence causes too many mapping biases. Other quantitative assays (RNA-Seq, small-RNA, ChIP-Seq, etc.) likely suffer from similar issues within individual samples but have not been systematically studied due to the high cost of creating individualized genome sequences *de novo*.

Nonetheless, a reference genome sequence is clearly needed for research. Without a point of reference and common coordinate, or naming system, research and clinical assay results cannot be reported in ways that allow for inter-lab comparisons and independent validation of research results. There are many important questions yet to be addressed as to how to best approach developing a universal reference sequence and establish best practices for using it. Addressing population and individual variability in a universal reference requires that we think about the genome, not as a single sequence, but rather as a union of differences. A basic coordinate system needs to be developed that can accommodate any indel and rearrangements, and analytical tools need to assume higher levels of differences than they do now. To begin addressing these issues, we need to have a much greater number of de novo assembled genomes from both evolutionarily distant and closely related individuals and improved methods for variant calling. Fortunately, much work is ongoing on both fronts[41]–[44], and the era of truly personalized medicine, which leverages an individually constructed genome, is on the horizon.

## Materials and Methods

### Data sources

1. The combined pilot data from the 1000 Genomes Project: ftp://ftp-trace.ncbi.nih.gov/1000genomes/ftp/release/20100804/ALL.2of4intersection.20100804.genotypes.vcf.gz
2. Release 23 of HapMap containing variant calls for 90 CEU individuals based on human reference assembly hg18.
3. The Complete Genomics data consisted of the 69 publicly available genomes that were released in April 2011 and were downloaded from: ftp://ftp2.completegenomics.com.
4. The list of GWAS studies from the UCSC browser [45] downloaded at: ftp://hgdownload.cse.ucsc.edu/goldenPath/hg19/database/gwasCatalog.txt.gz.

The 1KGP release includes data from 629 individuals and includes the variants identified by two of the four pipelines utilized by the 1KGP. Because of the variability between different software packages and a concern for false positives, the results of the four pipelines were merged to create a file including any call made by at least two of the pipelines. Further explanation of this process can be found at: ftp://ftp.1000genomes.ebi.ac.uk/vol1/ftp/release/20100804/README.20100804_genotypes_and_imputation. While this data from the 1KGP project is not the most current data, it is the only published data that is allowed for publishable analysis according to the Fort Lauderdale data release policies [46].

For the GC data, only called SNP genotypes were used, and no-call loci were ignored. All sequence data were aligned to hg19. The RefSeq genes release 37.1 was used for the determination of coding regions and the Complete Genomics annotations were used to identify of non-synonymous changes.

For the comparison of SNPs between the CG and 1KGP datasets, we took all of the hg19 coordinates for each genome, and then included a base-pair +1 and −1 for that location. This addition allowed for the potential single-base slippage [47] that often occurs in sequencing studies and the differences between 0-based and 1-based coordinates [45].

### Linkage Disequilibrium

The Linkage Disequilibrium (LD) analysis was performed with a combination of PLINK [48] and Golden Helix SVS package version 7.4 (Golden Helix Corp./Inc, Bozeman MT). First, both the HapMap and 1KGP datasets were trimmed using PLINK to only include variants with genotype calls in at least 99% of the alleles to prevent problems with missing data. Then, overall LD blocks for the samples were determined using the PLINK “–blocks” option for the entire genome to generate the summary statistics. Due to the tremendous amount of computational time that was required to perform these calculations, the sub-sampling of SNPs and individuals from the 1KGP data was done using chromosome 20 which is a good representative chromosome [49], [50] containing 2% of the human genome.

For the gene-specific analysis of BRCA1 and JAK2, the HapMap data were converted from hg18 to hg19 using the LiftMap tool (http://genome.sph.umich.edu/wiki/LiftOver). Next, the haplotype blocks were determined using Golden Helix SVS package version 7.4 (Golden Helix Corp./Inc, Bozeman MT) and the default settings for each of the two datasets for each gene. The “Haplotype Blocks” tables that were generated were then summarized into the reported results. The plots of LD were generated using the Plot Linkage Disequilibrium command with default settings. The Runs of Homozygosity (ROH) were determined using Golden Helix and the default parameters.

The determination of problematic microarray probes was made by querying the 1KGP annotation files against the reference files for the arrays provided by the vendors. The counts of variants within exons and genes were determined using the consensus coding sequence CCDS [51] genes and counting the number of variants found in the 1KGP annotation files. Polyallelic SNPs were determined separately for the CG and 1KGP files by combining the genotype calls for all of the individuals in each project. Any location in the genome having calls for more than two nucleotides across the individuals in a dataset was termed polyallelic.

## Supporting Information

Table S1 **A list of each of the GWAS studies from the UCSC database where we have identified a shortcoming with the probe underlying the published hit.** For each probe, we list the type of deficiency that has been identified.
(XLS)

Table S2 **A list of all microarray probes from the Illumina and Affymetrix platforms where we have identified a problem.**
(TXT)
